# Occult Breast Cancer due to Multiple Calcified Hamartomas in a Patient with Cowden Syndrome

**DOI:** 10.1155/2012/638725

**Published:** 2012-07-17

**Authors:** E. B. Gómez García, M. B. I. Lobbes, K. van de Vijver, K. Keymeulen, F. van der Ent, H. G. Yntema, V. C. Tjan-Heijnen, C. Boetes

**Affiliations:** ^1^Department of Clinical Genetics, GROW School for Oncology and Developmental Biology, Maastricht University Medical Centre, P.O. Box 5800, 6202 AZ Maastricht, The Netherlands; ^2^Department of Radiology, GROW School for Oncology and Developmental Biology, Maastricht University Medical Centre, P.O. Box 5800, 6202 AZ Maastricht, The Netherlands; ^3^Department of Pathology, GROW School for Oncology and Developmental Biology, Maastricht University Medical Centre, P.O. Box 5800, 6202 AZ Maastricht, The Netherlands; ^4^Department of Surgery, Maastricht University Medical Centre, P.O. Box 5800, 6202 AZ Maastricht, The Netherlands; ^5^Department of Surgery, Orbis Medical Center Sittard-Geleen, P.O. Box 5500, 6130 MB Sittard, The Netherlands; ^6^Department of Human Genetics, Radboud University Medical Center Nijmegen, P.O. Box 9101, 6500 HB Nijmegen, The Netherlands; ^7^Division of Medical Oncology, Department of Internal Medicine, GROW School for Oncology and Developmental Biology, Maastricht University Medical Centre, P.O. Box 5800, 6202 AZ Maastricht, The Netherlands

## Abstract

Cowden syndrome (CS) is an autosomal dominant disorder characterized by presence of multiple hamartomas, and other benign and malignant abnormalities of the breasts, skin, thyroid, endometrium, gastrointestinal tract, and central nervous system. Hamartomas are benign, developmentally disorganized tumors that can develop in any of the above mentioned organs. The presence of massive calcifications in the breasts in very young women is an indication to perform a breast MRI to exclude a neoplasm since, like in the current case report, presence of breast calcifications may obscure a neoplasm. Although fibrocystic disease and cooccurrence of fibrocystic disease and breast cancer are much more common than CS, the presence of massive calcifications in the breasts of very young women should elicit the possibility of an underlying genetic disease. Furthermore, breast cancer and macrocephaly are considered major criteria for the diagnosis of CS and the combination of both is enough to establish the clinical diagnosis of this entity. Fibrocystic disease of the breasts and multinodular goiter are minor criteria. Family history is also important for the diagnosis of (any) hereditary disease.

## 1. Case Report

**Figure 1:**
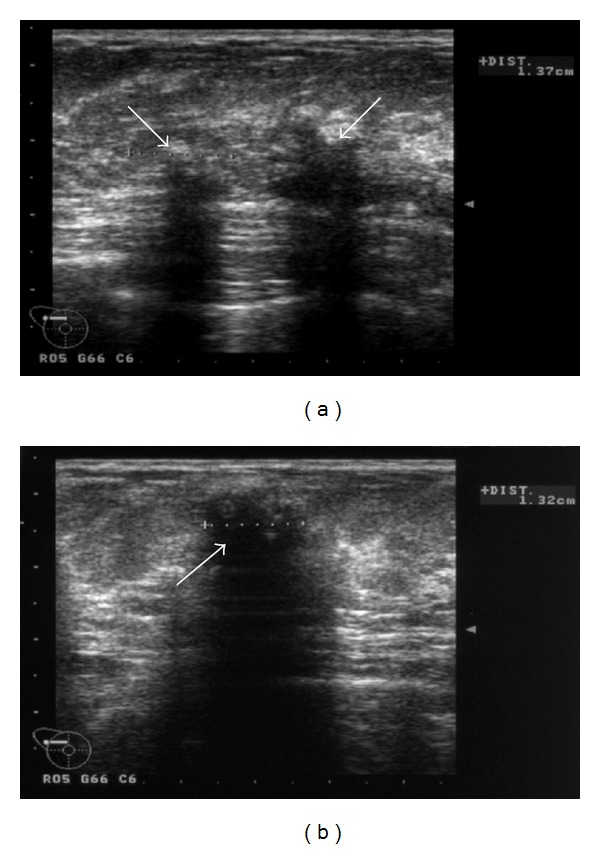
Ultrasound shows multiple, ill-defined hypoechoic areas with acoustic shadowing, indicating multiple coarse calcifications within the right breast (arrows).

**Figure 2:**
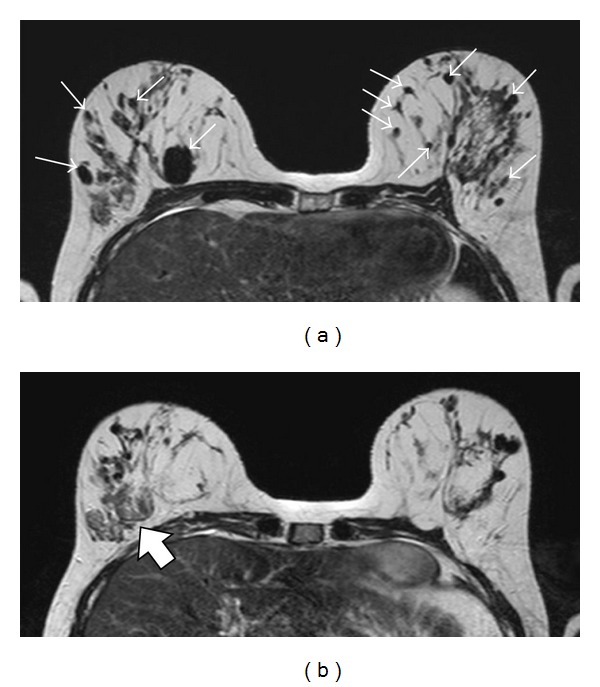
Transverse T2-weighted images show multiple coarse calcifications in both breasts ((a) small arrows) and a large, lobulated mass in the lower lateral quadrant of the right breast ((b) white arrow).

**Figure 3:**
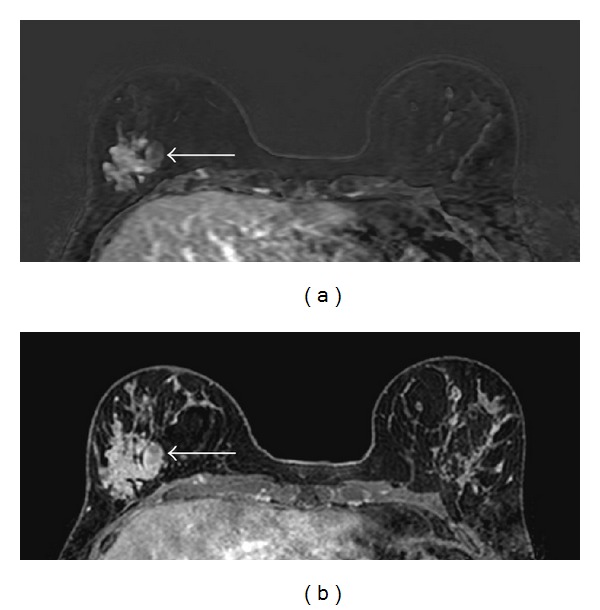
Contrast-enhanced (subtraction) MR images (a) and contrast-enhanced T1-weighted MR images (b) show a large, lobulated heterogeneously enhancing mass with irregular borders in the lower lateral quadrant of the right breast (arrows).

A 26-year-old woman was diagnosed with breast cancer from biopsy of an axillary lymph node. An ultrasound (Figure 1) followed by an MRI of the breasts (Figures 2 and 3) were subsequently performed. 

**Figure 4:**
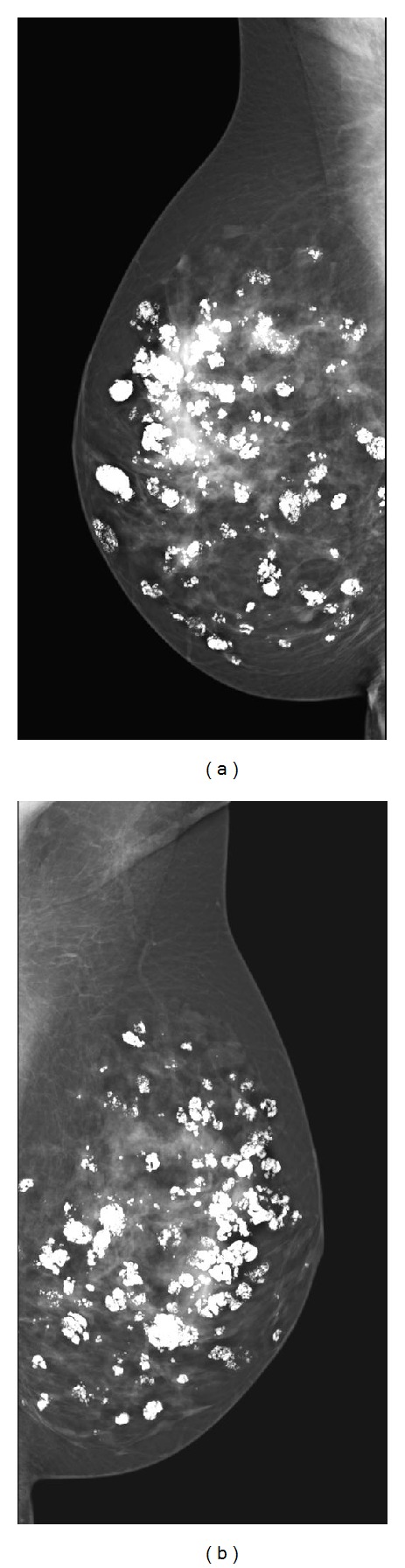
Patient's sister mammography (mediolateral oblique direction shown) showed massive calcifications in multiple, well-defined masses, some of them with adipose tissue, diagnostic of hamartomas.

The patient underwent annual breast ultrasound because of a family history of breast cancer. In addition, she had a history of multinodular goiter. By physical examination, it was also apparent that she had a relatively large head in relation to her length (length 170 cm and head circumference 61 cm, i.e., above 97th percentile). According to the patient, macrocephaly is a common feature in her family. Family history also revealed that the patient's mother had breast cancer at the age of 45. The patient's identical twin is known to have breast fibrocystic disease (Figure 4). 

In Figure 1, the ultrasound shows multiple, ill-defined hypoechoic areas with acoustic shadowing, indicating multiple coarse calcifications within the right breast. In Figure 2, transverse T2-weighted magnetic resonance images show multiple coarse calcifications in both breasts and a large, lobulated mass in the lower lateral quadrant of the right breast. Contrast-enhanced subtraction MR images (Figure 3(a)) and T1-weighted images (Figure 3(b)) show a large, macrolobulated enhancing mass with irregular margins in the lower lateral quadrant of the right breast. Finally, her sister's mammography (mediolateral oblique direction shown) showed massive calcifications in multiple, well-defined masses, some of them with adipose tissue, diagnostic of hamartomas.

Biopsy results showed that the patient had a locally advanced, invasive, well-differentiated ductal carcinoma in the right breast which was obscured on annual ultrasound due to massive calcifications by hamartomas. 

The radiological images, together with the thyroid pathology and macrocephaly, suggested Cowden syndrome (CS) as most likely underlying cause, which was later confirmed by identification of a germline splice-site mutation: c.634 + 2T > C in intron 6 of the *PTEN* gene. The mutation was inherited from the patient's mother.

The patient was treated with neoadjuvant chemotherapy with a partial response, followed by a modified radical mastectomy and preventive contralateral mastectomy, and by locoregional radiotherapy (because of 5 positive axillary lymph nodes) plus adjuvant endocrine therapy. 

## 2. Discussion

The presence of massive calcifications in the breasts in a very young woman is a plausible indication to perform an breast MRI to exclude a neoplasm since, like it was the case in our patient, the presence of breast calcifications may obscure a neoplasm. Although fibrocystic disease and co-occurrence of fibrocystic disease and breast cancer are much more common than CS (which has an estimated prevalence between 1/200,000 and 1/250,000 in the Dutch population [1]), the presence of massive calcifications in the breasts of very young women should elicit the possibility of an underlying genetic disease. Furthermore, breast cancer and macrocephaly are considered major criteria for the diagnosis of CS and the combination of both is enough to establish the clinical diagnosis of this entity [2]. Fibrocystic disease of the breasts and multinodular goiter are minor criteria [2]. Family history is also important for the diagnosis of (any) hereditary disease. 

Cowden syndrome, OMIM# 158350 [3], first described in 1963 by Lloyd and Dennis in a patient called Rachel Cowden [4], is also known as multiple hamartoma syndrome. It is an autosomal dominant disorder characterized by presence of multiple hamartomas, and other benign and malignant abnormalities of the breasts, skin, thyroid, endometrium, gastro intestinal tract, and central nervous system. Hamartomas are benign, developmentally disorganized tumors that can develop in any of the above mentioned organs [5, 6].

The National Comprehensive Cancer Network (NCCN) has established diagnostic criteria for CS, based on combinations of features classified as pathognomonic, major, and minor [2]. There are two pathognomonic criteria: a cerebellar tumor known as dysplastic gangliocytoma that causes a range of symptoms derived from compression, known as Lhermitte-Duclos disease, and the following mucocutaneous lesions: facial trichilemmomas, acral keratoses, and papillomatous nodules. Major criteria are: breast carcinoma, nonmedullary thyroid carcinoma, macrocephaly, and endometrial carcinoma. Minor criteria are: other thyroid lesions, mental retardation, gastrointestinal hamartomas, fibrocystic disease of the breast, lipomas, fibromas, tumors or structural malformations in the genitourinary tract and uterine fibroids.

Operational diagnosis of CS in an individual is done when the patient has any of the following: (1) presence of any pathognomonic lesion (by mucocutaneous lesions a certain number and/of combinations of these lesions are required); (2) two or more major criteria (but one has to be macrocephaly); (3) one major and three minor criteria, or (4) four minor criteria [2].

A mutation in *PTEN* (PTEN = phosphatase and tensin homologue), a tumor suppressor gene, is found in more than 80% of patients who fulfill these criteria [5]. So far, no genotype/phenotype correlations have been found among the patients with CS [1, 5].

In addition to CS, germline *PTEN* mutations have been associated with the following syndromes, globally known as *PTEN* hamartoma tumor syndromes (PHTS): Bannayan-Riley-Ruvalcaba syndrome (BRRS), Proteus, Proteus-like syndrome, adult Lhermite Duclos disease, and autism-like disorders associated with macrocephaly [6]. Of those entities, CS and BRRS (OMIM# 153480) [2] have the highest degree of clinical overlap, such as fibrocystic disease, macrocephaly, thyroid disease, lipomas, intestinal hamartomatous polyps, and mental retardation. BRRS is nowadays considered a variant of CS rather than a separate entity.

Breast cancer is the most frequent malignancy of CS. It occurs in 30–50% of patients, with an average age of diagnosis between 38 and 46 years, with the youngest reported case at age 14. Risk of bilateral breast cancer is estimated to be 25% [5, 6]. Like in the general population, ductal carcinoma is the most frequent histological type. However, the finding of hyalinized collagen surrounding the tumor is specific for CS [5]. Breast hamartomas are benign lesions and are often radiologically and morphologically indistinguishable from fibroadenomas. They are seen in areas of the breast with a combination of fat, fibrous, and muscular tissues, but, can also appear as ill-defined calcified masses suspicious of a malignancy [7]. Although hamartomas and carcinoma can colocalize, as it was the case in our patient, it is not clear whether the carcinoma arises from a hamartoma.

Although breast MRI examinations, only represent a small percentage of the MRI examinations performed, its use is growing rapidly. MRI is more sensitive than mammography in detecting tumors in women with an inherited predisposition due to mutation in *BRCA1*, *BRCA2*, *PTEN* or *TP53* genes [8]. However, MRI does not substitute mammography, that is, mammography is better to detect ductal carcinoma in situ and some small cancers. In 2007, the American Cancer Society published recommendations for breast MRI screening [9]. CS is one of the entities where there is enough evidence to recommend performing annual breast MRI in combination with mammography. Specific recommendations for women with CS include: semiannual physical breast examination, starting at age 25, annual mammography, and breast MRI beginning at age 30–35 or 5–10 years before the earliest diagnosis, as well as self-examination starting at age 18. Recommendations in The Netherlands [10] differ from those above in that women with CS are advised to undergo an annual MRI from age 25 and the annual mammogram is added from age 30. Prophylactic mastectomy is also an option for patients with CS and should be considered on a case-by-case basis. Both men and women are advised annual clinical examination of the thyroid gland, starting at age 18. 

In conclusion, when massive breast calcifications in mammograms of young women are observed, the diagnosis of CS has to be considered. A breast MRI is indicated in those cases to exclude a neoplasm.
